# Phosphorus-Containing Polybenzoxazine Aerogels with Efficient Flame Retardation and Thermal Insulation

**DOI:** 10.3390/ijms24054314

**Published:** 2023-02-21

**Authors:** Yusheng Que, Chunxia Zhao, Jixuan Wei, Fahong Yang, Hui Li, Jinbo Cheng, Dong Xiang, Yuanpeng Wu, Bin Wang

**Affiliations:** 1 School of New Energy and Materials, Southwest Petroleum University, Chengdu 610500, China; 2 The Center of Functional Materials for Working Fluids of Oil and Gas Field, Sichuan Engineering Technology Research Center of Basalt Fiber Composites Development and Application, Southwest Petroleum University, Chengdu 610500, China

**Keywords:** polybenzoxazine, 10-(2, 5-dihydroxyphenyl)-10- hydrogen-9- oxygen-10- phosphine-10- oxide (DOPO-HQ), composite aerogel, flame retardancy, thermal insulation

## Abstract

Bisphenol A type benzoxazine (Ba) monomers and 10-(2, 5-dihydroxyphenyl)-10- hydrogen-9- oxygen-10- phosphine-10- oxide (DOPO-HQ) were employed to prepare flame retardant and heat insulated polybenzoxazine (PBa) composite aerogels. The successful preparation of PBa composite aerogels was confirmed by Fourier transform infrared (FTIR), X-ray photoelectron spectroscopy (XPS), and scanning electron microscopy (SEM). The thermal degradation behavior and flame-retardant properties of the pristine PBa and PBa composite aerogels were investigated with thermogravimetric analysis (TGA) and cone calorimeter. The initial decomposition temperature of PBa decreased slightly after incorporating DOPO-HQ, increasing the char residue amount. The incorporation of 5% DOPO-HQ into PBa led to a decrease of 33.1% at the peak of the heat-release rate and a decrease of 58.7% in the TSP. The flame-retardant mechanism of PBa composite aerogels was investigated by SEM, Raman spectroscopy, and TGA coupled with infrared spectrometry (TG-FTIR). The aerogel has advantages such as a simple synthesis procedure, easy amplification, lightweight, low thermal conductivity, and good flame retardancy.

## 1. Introduction

It is well established that aerogels are solid materials with a three-dimensional nanoporous network structure, lightweight, high surface areas, and ultralow thermal conductivity. Aerogels have incomparable advantages in flame retardation, heat insulation, electromagnetic shielding, and other fields. Polybenzoxazine (PBa) is a kind of thermosetting resin with excellent mechanical strength and thermal stability, as well as nearly zero volume shrinkage and no volatiles releasing during the ring opening cross-linking process of benzoxazine (Ba) monomers [1,2,3,4,5,6]. It can provide a platform to stack basic units and tune desired aerogel properties.

The development, application, and problems of conventional flame-retardant methods were outlined in recent years [7]. It is worth noting that some researchers achieve flame retardant effects by constructing unique oxazine ring structures by synthesizing backbone dihydroxy-deoxybenzooxazine-benzooxazine polymers [8,9] and the tetrafunctional self-polymerization. In this process, cyclosiloxane, as the core, forms benzoxazine compounds [10], which are halogen-free and phosphorous-free flame-retardant benzoxazine materials. Nevertheless, in order to achieve high flame retardation, a large number of flame retardants were added. Phosphorus compounds [11,12,13] or silicon-oxygen compounds are commonly added to improve flame retardancy [14,15].

Currently, there is a search for more materials that are lighter in weight and perform better. Previously, ring-opening polymerization of benzoxazine in solvents led to gelation and the formation of 3D polymer networks [16,17,18]. The aerogel drying process is considered the most critical step in aerogel production because of its ability to preserve the three-dimensional pore structure, leading to unique material properties such as high porosity, low density, and large surface area. Supercritical drying [19,20,21] and low-temperature freeze-drying are commonly used drying methods. Different drying methods also have a particular impact on the properties of aerogels [22]. Some studies have reported polybenzoxazine aerogels blankets prepared by supercritical drying method [23,24], using various sodium montmorillonite (Na-MMT) colloidal dispersions in chitosan/benzoxazine polymerization. In those studies, the aerogel was obtained by freeze-drying in an aqueous mixture solution [25]. As a new type of organic aerogel, polybenzoxazine aerogels can be dissolved in an organic solvent. So, the drying process does not need to use the supercritical method and low-temperature freeze drying [26], which dramatically reduces equipment pressure. Lightweight, strong, and thermally insulating polymethylsilsesquioxane-polybenzoxazine aerogels prepared by the sol-gel method and atmospheric drying were reported [27]. Some scholars used hydrochloric acid to form highly photoreactive carbon aerogels through ring-opening at room temperature after intercalation into C3N4 [28]. The effect of curing temperature on the properties of polybenzoxazine aerogel was discussed in the literature [29]. There are experiments that synthesized polybenzoxazine aerogels through room temperature ring-opening polymerization and atmospheric drying technology using an oxalic acid catalyst [30].

The aerogel structure can be stabilized further, which has many applications in hydrophobicity and improvement of mechanical strength [31,32]. In addition, polybenzoxazine aerogels have excellent compatibility, and many reports have studied bio-based benzoxazine blends to make aerogels [33], including the blends cross-linked with nanofibers. A new polybenzoxazine aerogel with three-dimensional porous materials was successfully prepared by blending benzoxazine with graphene oxide nanosheets. It was shown that the functional groups on graphene oxide and benzoxazine were in the process of heat treatment. Interactions in 3D are essential for forming 3D network structures [34]. If possible, low cost and an easy fabrication process are desirable.

In this study, multifunctional aerogels with high flame retardancy and low thermal conductivity are prepared through a sol-gel method using Ba as a reaction monomer, hydrochloric acid as a catalyst for ring-opening benzoxazine monomers, and DOPO-HQ as a flame retardant. DOPO-HQ effectively enhanced the flame-retardant properties of the PBa aerogels. This work provides cost-effective and environmentally friendly strategies for manufacturing flame-retardant polymer aerogels.

## 2. Results and Discussion

### 2.1. Chemical Structure of PBa and PBa Composite Aerogels

**Figure 1:**
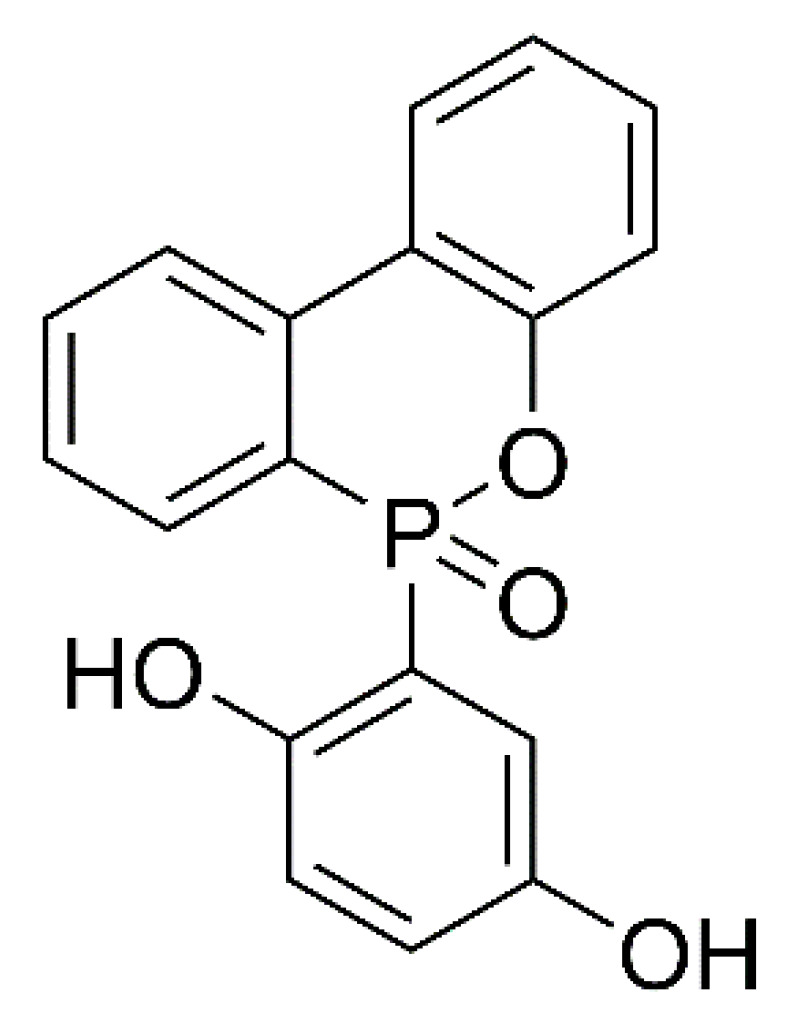
The Structural Formula for DOPO-HQ(DQ).

**Figure 2:**
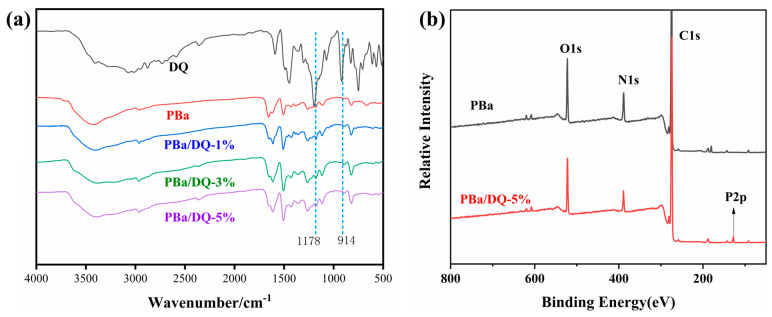
(**a**) The FITR Spectroscopy and (**b**) XPS of Aerogels.

The structural formula for DOPO-HQ(DQ) is shown in Figure 1. In Figure 2a, the characteristic absorption peaks of DOPO-HQ and other samples were analyzed by FTIR. In the FTIR spectra the characteristic absorption peaks near 1178 and 914 cm^−1^ are attributed to the P-O-Ar, and the absorption peak near 1450 cm^−1^ corresponds to the P- Ar vibrations. It is observed that the absorption peaks of P-O-Ar and P- Ar are enhanced with the increase in DOPO-HQ content. In addition, all aerogels show broad O-H absorption peaks around 3420 cm^−1^, which is due to the ring opening of benzoxazine. The characteristic absorption peaks in the range of 1580–1700 cm^−1^ are due to the motion of the benzene ring caused by the C=C of the skeleton [35,36,37]. Figure 2b presents the XPS spectra of PBa and PBa/DQ-5% aerogel. It shows that the main elements of PBa and PBa/DQ-5% are O, N, and C. Compared with PBa, PBa/DQ-5%, the characteristic peaks around 133–134 eV correspond to the phosphorus element in DOPO-HQ. The above discussion suggests the successful preparation of PBa composite aerogels.

### 2.2. Surface Morphology of PBa and PBa Composite Aerogels

**Figure 3:**
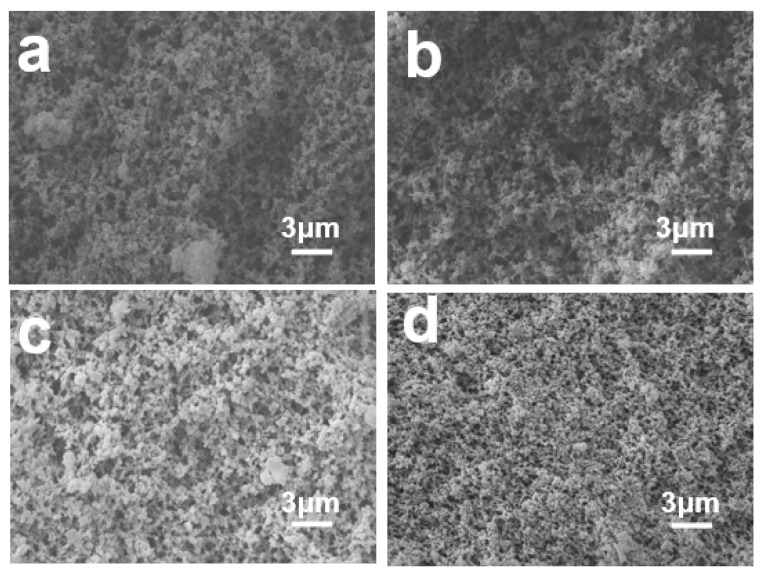
SEM Images of (**a**) PBa; (**b**) PBa/DQ-1%; (**c**) PBa/DQ-3%; and (**d**) PBa/DQ-5%.

PBa aerogels of various shapes and sizes can be obtained by sol-gel and atmospheric drying methods. The porous surface morphologies of PBa, PBa/DQ-1%, PBa/DQ-3%, and PBa/DQ-5% aerogels were investigated by SEM shown in Figure 3. The scanning electron microscopy images revealed that the corresponding aerogels consist of spherical polymer particles as the building block. It can be seen that all aerogels have a 3D network structure. However, the pore sizes are not uniform and are mainly distributed in the 10–100 nm range. However, with the increase in DOPO-HQ monomer concentration, the porous structure gradually becomes dense.

**Figure 4:**
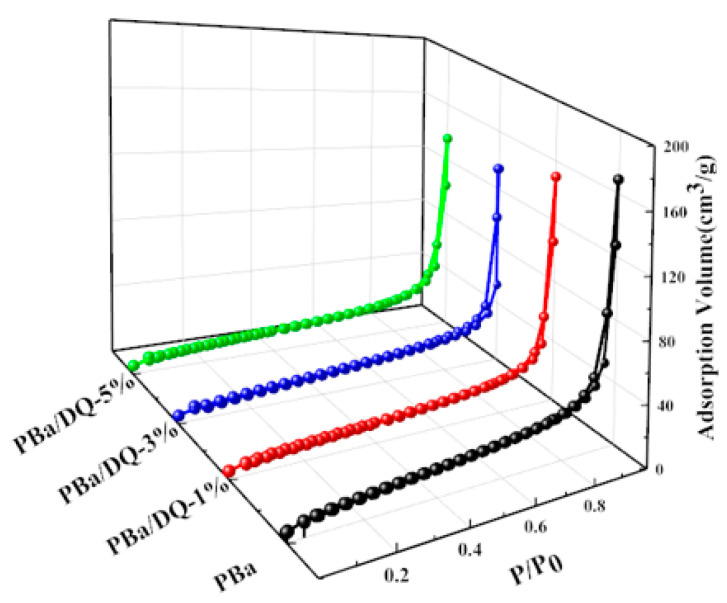
Nitrogen adsorption-desorption isotherms of aerogels.

**Figure 5:**
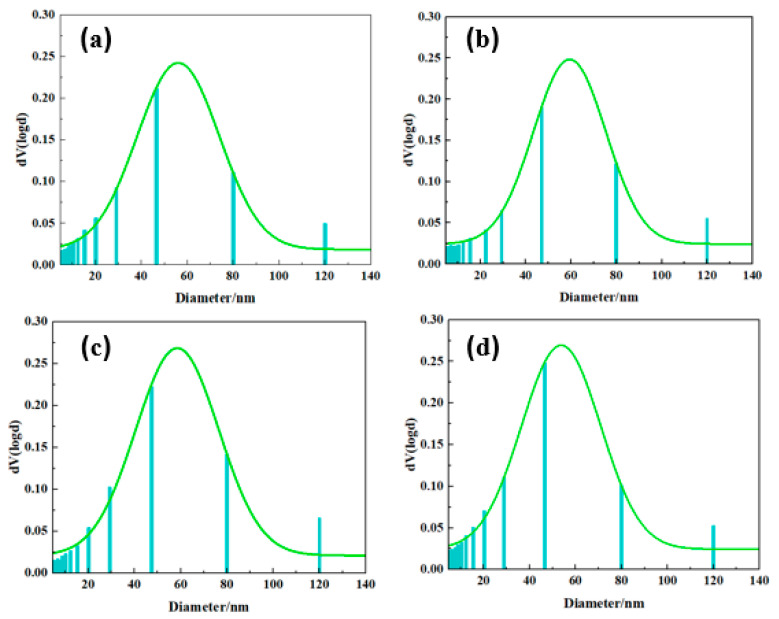
Pore distributions of different aerogels: (**a**) PBa; (**b**) PBa/DQ-1%; (**c**) PBa/DQ-3%; (**d**) PBa/DQ-5%.

The porous structure of the aerogel was further probed using nitrogen adsorption techniques. As shown in Figure 4, all aerogels exhibit typical IV isotherms with visible hysteresis loops. The isotherm rises sharply at P/P0 = 0.8–0.9, indicating abundant mesopores and few macropores in the PBZ aerogel [38,39,40]. The BET surface areas of PBa, PBa/DQ-1%, PBa/DQ-3%, and PBa/DQ-5% were 48, 50, 51, and 53 m^2^ g^−1^, respectively. The pore size distribution map in Figure 5 shows that all PBa aerogels have a porous structure concentrated in the range of 10–150 nm. It is consistent with the pore structural features shown by the adsorption-desorption isotherms.

### 2.3. Thermal Insulation Performance of PBa and PBa Composite Aerogels

**Figure 6:**
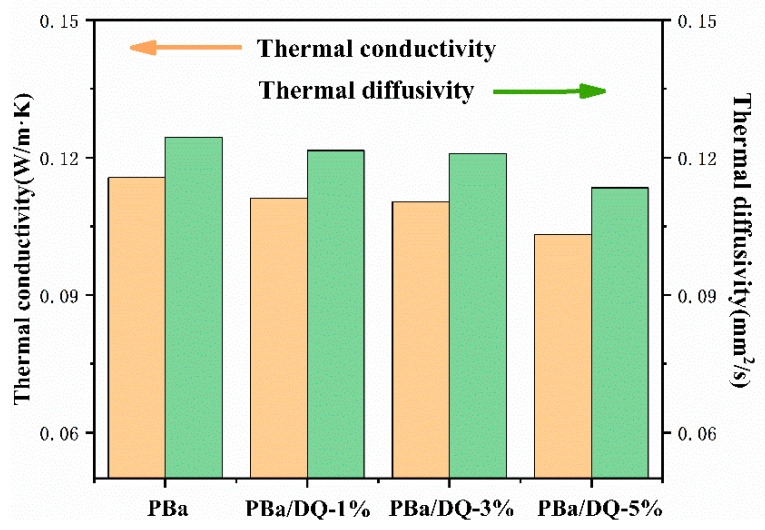
The thermal conductivity and thermal diffusivity of aerogels.

The thermal insulation performances of aerogels are shown in Figure 6. The thermal conductivity and thermal diffusivity have been tested, respectively. Thermal diffusivity refers to a physical quantity that changes the temperature of an object after a specific heat gain or loss. It can be seen from Figure 6 that with the increase in DOPO-HQ content, the thermal conductivity and thermal diffusivity of the aerogel decrease. It indicates that the addition of DOPO-HQ is beneficial in improving the thermal insulation performance of the aerogel. A large number of pores and uniform pore distribution can effectively inhibit gas heat transfer and slow the heat transfer rate [41,42].

### 2.4. Thermal Degradation Behaviors of PBa and PBa Composite Aerogels

**Figure 7:**
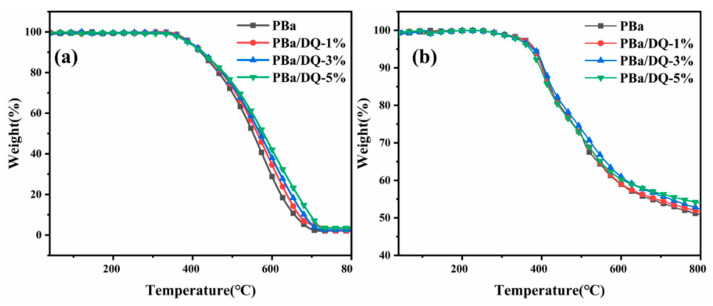
TGA of aerogels in the (**a**) Air; and (**b**) Nitrogen.

The thermal degradation behaviors of PBa and PBa composite aerogels were analyzed through TGA (Figure 7). The onset decomposition temperature T_onset_ is the temperature at which the mass loss is 5%. The temperature T_max_ is the temperature at which the maximum mass loss rate occurs. The amount of carbon residue is shown in Table 1. After incorporating DOPO-HQ, the residual carbon content of aerogels at 800 °C was significantly improved in both air and nitrogen atmospheres. The T_onset_ of PBa/DQ-1% in the air decreased from 392 °C.

Similarly, for PBa, the T_onset_ decreased to 390 °C. Finally, for PBa/DQ-1%, T_onset_ decreased from 382 °C to 380 °C in the N_2_ atmosphere. Compared with PBa, the T_onset,_ and T_max_ of PBa/DQ-5% composite aerogels decreased by 6 °C and 10 °C in air. Moreover, the T_onset_ and T_max_ decreased in the N_2_ atmosphere by 11 °C and 4 °C. It is because DOPO-HQ contains a phosphorus group, which is more unstable than the carbon chain skeleton structure of the PBa matrix. This phosphorus group decomposes quickly and captures the water in the air to make acid. This process promotes the dehydration and carbonization of the matrix, which forms on the surface of the matrix. The density of the carbon layer prevents the exchange of heat and substances between the interior of the polymer and the surrounding environment [43,44,45,46].

From Table 1 and Figure 7, it can be seen that the residue of PBa is significantly increased with increasing DOPO-HQ in the temperature range of 500–700 °C in air, whereas in nitrogen, this increase is not that significant. This may indicate oxidation resistance of the developing/developed char, and hence better fire retardance of the composite aerogels. Above 700 °C, this effect is minimal, as seen from residue at 800 °C [47,48]. When the addition of DOPO-HQ is 5%, the carbon residue in the air can reach 8.8% at 700 °C and 3.4% at 800 °C. A slight increase in residual carbon content was observed in both air and nitrogen. Consequently, incorporating DOPO-HQ in PBa increased from 1% to 5%. It means that DOPO-HQ makes the carbon residue of the composite aerogel denser and complete during the PBa combustion process. The thermal decomposition reaction of PBa and PBa composite aerogels in the air is the same as in N_2_ [49]_._ The addition of DOPO-HQ can slow down the combustion reaction of the material, increase the carbon residue rate of the polymer, and improve its resistance to flames.

PBa and its composite aerogels mainly occur between 300 and 500 °C. The less stable O=P-O bonds in DOPO-HQ can decrease the initial decomposition temperature of PBa and PBa composite aerogels. When temperatures exceed 300 °C, DOPO-HQ can produce phosphoric acid or polyphosphoric acid, H_2_O, and other substances. These substances are conducive to forming stable carbon residues, reducing heat and material transfer, and preventing further decomposition.

### 2.5. Flammability Performance of PBa and PBa Composite Aerogels

**Figure 8:**
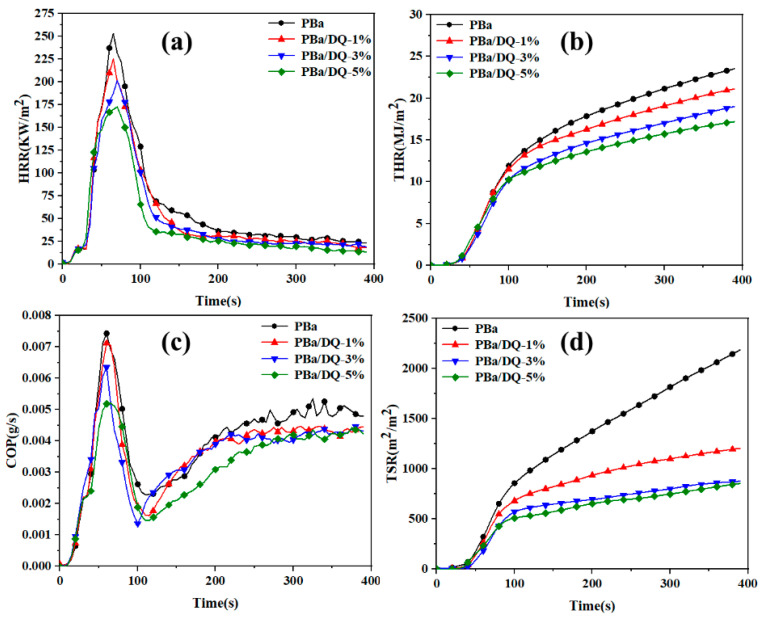
(**a**) HRR; (**b**) THR; (**c**) COP; and (**d**) TSR versus time curves of PBa and PBa composite aerogels obtained from the cone calorimetry test.

The cone calorimeter is a fire test instrument capable of evaluating the combustion characteristics and potential fire safety of polymers. Cone calorimetry experiments were used to evaluate the combustion properties of PBa composite aerogels and high-temperature cured benzoxazine solid slab (PBr). All samples were cut to 100 mm × 100 mm × 3 mm in size for the CONE test. The heat release rate (HRR), total heat release rate (THR), carbon monoxide production (COP), and total smoke release rate (TSR) are shown in Figure 8. Table 2 gives the corresponding time to ignition (TTI), peak heat release rate (PHRR), average heat release rate (AvHRR), and total smoke release rate (TSR). The cone calorimeter of the PBr is shown in Figure S1.

Time to ignition (TTI) was used to assess the effect of DOPO-HQ on the flammability of composite aerogels. It can be seen from Table 2 that the TTI value of PBr is 86 s, which is much larger than that of PBa and PBa composite aerogels. With the increase in DOPO-HQ content, the TTI value of PBa composite aerogels shows a tendency to taper off. In particular, the TTI value of PBa/DQ-5% is only 27 s, much lower than the 35 s of PBa. The ratio TTI/PHRR is used to evaluate the potential hazard of polymer materials. The higher the ratio, the lower the fire risk. The P-O and P=O in DOPO-HQ lead to shortened TTI and improved fire resistance of the material. The AvHRR of the composite aerogels decreased significantly with an increasing amount of DOPO-HQ added to the polymer (Table 2). The AvHRR of PBa/DQ-5% is 46.6 kW/m^2^, which is 27.9% lower than that of PBa. The lower AvHRR value of DOPO-HQ in PBa composite aerogel can promote the formation of a dense protective layer on the material’s surface, reducing the possibility of a combustion reaction. In Figure 8 and Table 2, the PHRR value of PBa/DQ-5% is significantly low. Specifically, the PHRR of PBa/DQ-5% is 172.1 kW/m^2^, which is 33.1% lower than that of PBa and 44.9% lower than that of PBr. The significant reduction in PHRR is mainly because the introduction of DOPO-HQ into the PBa composite aerogel promotes the formation of a cross-linked structure in the polymer matrix and a dense and compact carbon layer on its surface. That is why composite aerogel has better fire performance.

Figure 8b shows the THR curve of the PBa and PBa composite aerogels. Table 2 shows that the PBa/DQ-5% led to a 79% reduction in THR for PBr. Compared with PBa, the THR value of PBa/DQ-5% decreased from 23.3 MJ/m^2^ to 17.1 MJ/m^2^, indicating that the addition of flame retardants could reduce the heat release rate of benzoxazine. The introduction of DOPO-HQ can create a physical barrier that helps reduce mass transfer and fire hazards during combustion. It is worth noting that smoke is more deadly than burning. The COP value can assess the toxicity released by the material during combustion. Compared with PBa, the COP value of the composite aerogel with 5% DOPO-HQ content was significantly reduced, as shown in Figure 8c. Adding DOPO-HQ can significantly reduce CO in the air during the combustion of PBa and PBa composite aerogels. Therefore, the toxicity of the surrounding environment is reduced [50].

Moreover, in the combustion reaction process, the smoke generation is mainly caused by incomplete combustion. In addition, the decrease in TSR and TSP values means that the addition of DOPO-HQ has a significant smoke suppression effect on the PBa composite aerogel. The PBa/DQ-5% results in a 59% reduction in TSP for PBa, reducing the risk of suffocation for trapped persons in the fire [51]. Therefore, as a flame retardant, DOPO-HQ can effectively improve the flame retardancy of PBa aerogels.

### 2.6. Analysis of Flame Retardancy Mechanism

**Figure 9:**
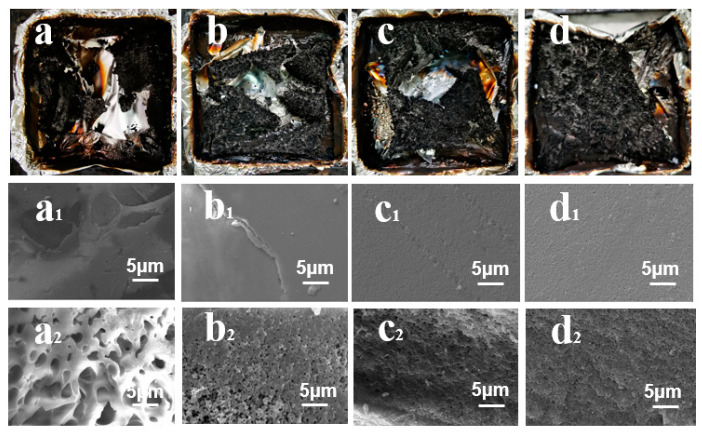
Digital photographs of the (**a**–**d**) residual chars and (**a_1_**–**d_1_**) SEM images of the residual char surfaces and (**a_2_**–**d_2_**) interior regions of (**a**) pure PBa; (**b**) PBa/DQ-1%; (**c**) PBa/DQ-3%; and (**d**)PBa/DQ-5%.

SEM was performed to investigate the morphology of the residual carbon for pure PBa and PBa composite aerogels, as shown in Figure 9. First, as can be seen from the digital photos of the pure PBa carbon residue, the carbon layer is very thin and loose. However, the carbon layer becomes thicker and denser with the addition of DOPO-HQ. In particular, the carbon layer formed by PBa/DQ-5% is thick, strong, and continuous. Moreover, because the surface of the composite forms a dense carbon layer, it can effectively isolate the exterior. The flame retardant PBa matrix prevents the heat from entering the bottom layer. As shown in Figure 9a, it can be observed that the pores are dispersed and non-uniform. In addition, the pores formed in pure PBa are large. Hence, the flame retardancy of pure PBa is poor. This conventional carbonized layer is brittle and vulnerable. So, pure PBa is less protective against gas and heat.

Figure 9(a_1_–d_1_) shows that the structure of the surface is compact and continuous by PBa/DQ-5% compared to pure PBa. This structure can prevent the escape of small molecules of combustible gas and the heat transfer of the burning surface and the material under the carbon layer. Figure 9(a_2_–d_2_) shows that adding DOPO-HQ increases the internal porous structure. Figure 9d shows a good dispersion state in PBa/DQ-5% pores. It indicates that due to the addition of DOPO-HQ, the composite surface forms an effective barrier to prevent the oxygen, heat, and mass transfer between the combustion zone and the inner zone, improving the flame retardancy of the composite.

It can be seen from Figure 9 below that with the increase in the amount of DOPO-HQ, a high-quality carbon layer is gradually formed. This layer blocks heat, air, and hydrolyzed products, giving a flame-retardant effect in PBa compounds. Moreover, with the increase in DOPO-HQ, it can be seen that the pore size is continuously reduced, and the distribution is uniform [52].

**Figure 10:**
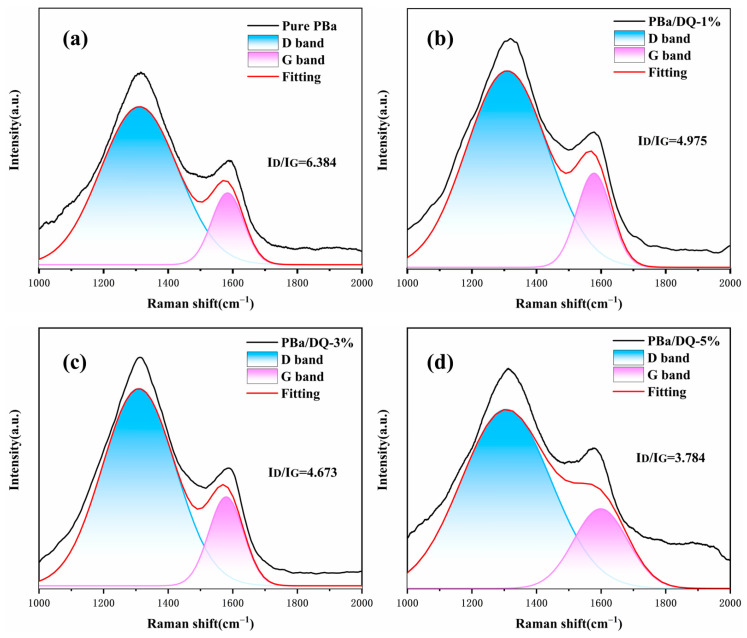
Raman spectra of charred residue of (**a**) PBa; (**b**) PBa/DQ-1%; (**c**) PBa/DQ-3%; (**d**) PBa/DQ-5%.

Furthermore, Raman spectroscopy was employed to measure the chemical structure of the char residue obtained from cone calorimetry tests. The degree of graphitization is the ratio ID/IG, reflecting the defect level or integrity of carbonaceous materials. When materials decompose, a higher graphitization of the char layer indicates a remarkably restrained performance on O_2_ and mass transfer. As shown in Figure 10, PBa/DQ-5% char shows a minimum value of ID/IG corresponding to a high graphitization degree. It reveals that the char residue of PBa and PBa composite aerogels are much more structurally integrated than the pure PBa [53]. This property is consistent with our previous SEM observations.

### 2.7. Gas Phase Analysis

**Figure 11:**
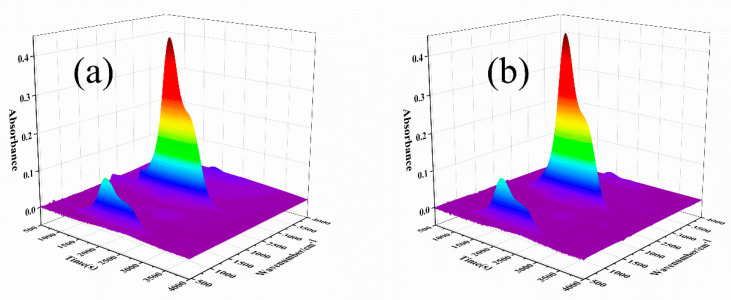
Three-dimensional TG-FTIR images of pyrolysis products of (**a**) PBa; (**b**) PBa/DQ-5%.

**Figure 12:**
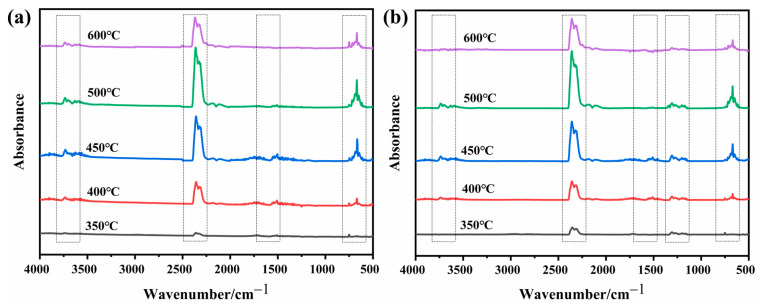
FTIR spectra of (**a**) PBa; (**b**) PBa/DQ-5% at different temperatures.

**Figure 13:**
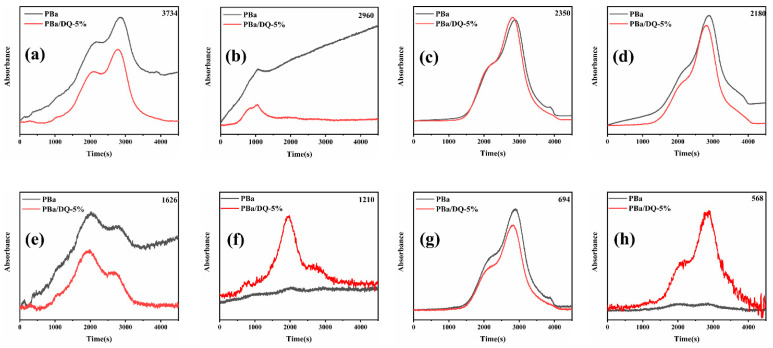
The analysis of pyrolysis products for pure PBa, PBa/DQ-5%: (**a**) water and ammonia gas; (**b**) alkane-containing compounds; (**c**) CO_2_; (**d**) CO; (**e**) and (**g**) aromatic compounds; (**f**) and (**h**) P–O and P=O compounds.

In order to study the role of D-fu in the gas phase during combustion, the pyrolysis products of PBa and PBa composite aerogels were analyzed by the TG-FTIR technique. Three-dimensional (3D) TG-FTIR spectra were recorded during the thermal decomposition process, as shown in Figure 11. The TG-FTIR profiles of PBa/DQ-5% and PBa are similar. Specifically, the TG-FTIR spectra of the primary pyrolysis products of pure PBa are located in the ranges 3700–3800 cm^−1^, 2900–3000 cm^−1^, 2350 cm^−1^, and 1500–1750 cm^−1^ corresponding to H_2_O and NH_3_, alkane, carbon dioxide, and aromatic compounds, respectively. In Figure 12a,b, P-O, and P=O groups in DOPO-HQ undergo thermal degradation at lower temperatures. At the same time, it is evident that the introduction of DOPO-HQ plays a crucial role in improving the pyrolysis of raw materials. Figure 13a–h shows the characteristic peak intensity of FTIR spectra of decomposition products with time. Firstly, the intensity of characteristic peaks increases with the addition of DOPO-HQ, as shown in Figure 13a. The production of water and ammonia gas causes it. At the same time, the intensity of CO_2_ peak in the TG-FTIR spectrum of PBa/DQ-5% composite increased in Figure 13c. These non-flammable gases weaken the fuel and O_2_ concentrations in the gas phase, thereby inhibiting combustion to some extent. In addition, the peaks in Figure 13b,d,e,g correspond to alkane compounds, CO, and aromatic compounds, respectively. In addition, the PBa/DQ-5% polymer produces fewer unstable decomposition compounds than pure PBa. These results indicate that adding DOPO-HQ can improve the flame retardancy of the PBa matrix [54,55].

Most importantly, the peaks can be attributed to the tensile vibrations of the P-O and P=O bonds, respectively (Figure 13f,h). These bands imply that some phosphorus-containing groups in DOPO-HQ are degraded into gaseous components during thermal decomposition. In the gas phase, the continuous combustion of polymer materials is responsible for forming H and HO radicals. DOPO-HQ in PBa matrix generates PO radicals during combustion and can capture HO and H radicals in the flame region. In conclusion, the significant improvement of the flame retardancy of PBa/DQ-5% composites can be attributed to the physical inhibition of the carbonized layer by the flame retarding system containing P and the dilution of non-flammable gases during combustion.

**Figure 14:**
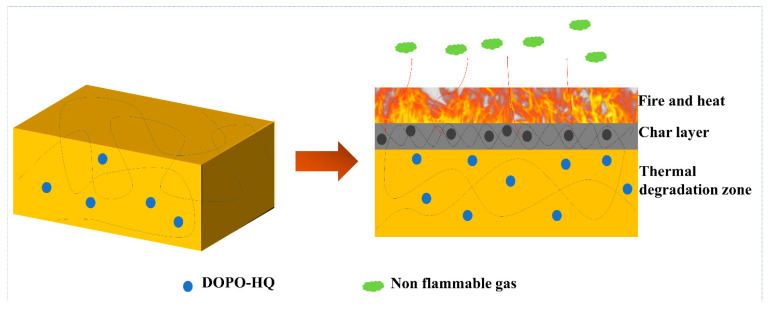
Possible flame-retardant mechanism.

The speculation on FR for the PBa/DOPO-5% composite is illustrated in Figure 14. Once ignition takes place, the DOPO begins to produce char residues due to the highly condensed phase while producing PO radicals that starve the flame of combustible volatiles [56]. The graphitic content char produced by the PBa/DQ-5% composite begins to impede the transfer of heat and oxygen inside the composite, thereby reducing the intensity of the fire. During this process, the porous structure collapses, which further enhances the flame retardancy causing a decrease in the amount of CO and smoke particles. Hence, PBa composite aerogels FR combines the effect of solid and gas phase actions to reduce the intensity of fires and, at the same time, suppress smoke and other volatile/toxic gases.

## 3. Materials and Methods

### 3.1. Materials

Bisphenol A type benzoxazine (Ba) was provided by Sichuan Tiance Jucai Technology Co., Ltd. 10(2,5-Dihydroxypheny)-10h-9-oxa-10-phospha-phenanthrene-10-oxide (DOPO-HQ) was purchased from Shanghai Xushuo Biotechnology Co. Ltd., (Shanghai, China). N, N-Dimethylamide (DMF), and ethanol were bought from Chengdu Kelong Chemical Reagent Factory (Chengdu, China). All chemicals were used as received without further purification.

### 3.2. Preparation of Benzoxazine/DOPO-HQ Mixed Solution

First, 5 g of bisphenol A benzoxazine and 40 g of DMF were added to a three-necked flask (250 mL). The mixture was stirred at room temperature to dissolve the benzoxazine monomer completely. After that, 1.25 g of concentrated hydrochloric acid was added and stirred for 60 min under ice bath conditions. After the solution turned from light to dark yellow, 0.05 g of DOPO-HQ (0.01 mol) was added and stirred until fully dissolved. The benzoxazine mixed solution was finally obtained.

### 3.3. Preparation of Polybenzoxazine/DOPO-HQ Aerogels

**Figure 15:**
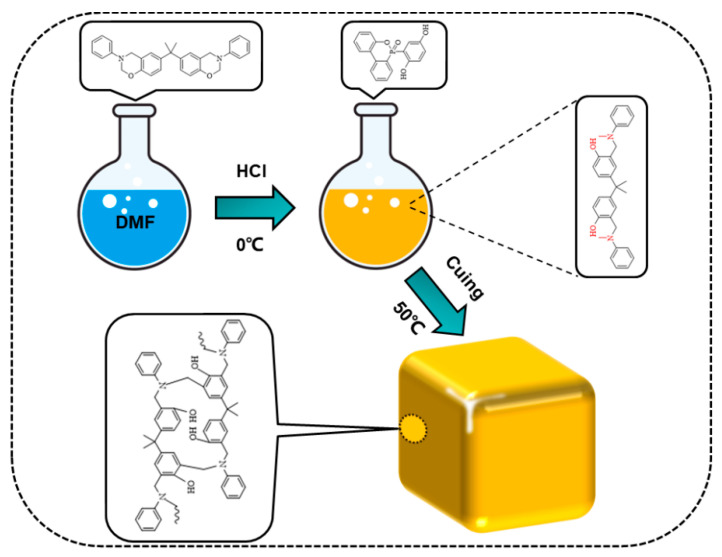
The preparation process of PBa composite aerogels.

First, the mixed benzoxazine/DOPO-HQ was poured into the mold and put in a constant-temperature oven. It was aged at 50 °C for 72 h to solidify. After that, the wet gel was removed and soaked in absolute ethanol for 72 h. The absolute ethanol was replaced every 12 h to replace the DMF in the wet gel. After removing DMF, the wet gel was dried at normal temperature and pressure, and finally, a composite aerogel of PBa/DQ-1% was obtained. Similarly, PBa, PBa/DQ-3%, and PBa/DQ-5% aerogels were prepared according to the same experimental procedure, as shown in Figure 15.

Polybenzoxazine panel (PBr) as a control sample was prepared through the following route. A total of 80 g of Ba was melted and poured into a glass mold and pre-cured at 180 °C/2 h and further reacted at 200 °C/2 h. The final material was marked as PBr. The thickness of PBr, PBa, and PBa/DQ samples was all around 3 mm.

### 3.4. Characterization

The pulverized aerogel surface functional groups were tested by FTIR spectroscopy in the 4000–500 cm^−1^ range using a Nicolet 6700 spectrometer (Thermo Electric Corporation, Waltham, MA, USA). The X-ray photoelectron spectrometer model used in this study was Nexsa from Thermo Scientific. The aerogel surface morphologies and carbon layers were analyzed with a scanning electron microscope (SEM) ZEISS EV0 MA15. Aerogel pore size distribution was assessed using a Mike ASAP2460, the adsorbed gas is nitrogen, degassed at 120 °C for 8 h. The thermal insulation properties of the aerogels were tested using a Hot Disk TPS 2500 S. TGA was performed using a DSC823 TGA/SDTA85/e from 40 °C to 800 °C at a ramp rate of 10 °C/min in nitrogen and air. The fire retardance performance of polymer materials were evaluated by cone calorimetry with ASTM E1354/ISO 5660. The samples were 100 mm × 100 mm × 3 mm in size, encapsulated in aluminum foil, and measured at a heat flow of 35 kw/m^2^. The structure of char residue from the cone calorimetry test was analyzed using Raman spectroscopy (Hrobia Xplra plus Raman) equipped with a laser wavelength of 785 nm. Thermogravimetric analysis coupled with Fourier transform infrared (TG-FTIR) was conducted with a Perkin-Elmer STA 6000 in nitrogen and air atmospheres with a heating rate of 10 °C/min from 40 °C to 800 °C.

## 4. Conclusions

Various methods were employed to analyze the effect of DOPO-HQ on the micro-structure properties such as thermal degradation, flame retardance, and heat insulation. This work successfully fabricated PBa composite aerogels with excellent flame-retardant properties. PBa/DQ-5% aerogel has low thermal conductivity and good thermal insulation performance because of its uniform pore size. Compared with PBa aerogel, coke production increased at 700 °C and 800 °C with DOPO-HQ aerogel. In addition, cone calorimetry, Raman spectroscopy, and TG-IR tests confirmed that the addition of DOPO-HQ achieved aerogel flame retardancy and reduced the production of toxic gases and smoke. PBa/DQ aerogel has a potential application prospect in the field of high flame retardation requirements.

## Figures and Tables

**Table 1:** Results of thermal analysis of PBa and PBa composite aerogels in air and nitrogen.

Sample	T_onset_ (°C)		T_max_ (°C)		Char Residue(%)			
					700 °C		800 °C	
	Air	N_2_	Air	N_2_	Air	N_2_	Air	N_2_
PBa	392	382	521	405	2.7	54.0	2.0	50.9
PBa/DQ-1%	390	380	520	403	5.1	54.6	2.1	51.7
PBa/DQ-3%	389	377	517	403	5.2	55.8	2.4	52.4
PBa/DQ-5%	386	371	511	401	8.8	56.5	3.4	53.9

**Table 2:** CONE parameters of PBr, PBa, and PBa composite aerogels.

Sample	TTI (s)	PHRR (kW/m^2^)	AvHRR (kW/m^2^)	THR (MJ/m^2^)	TSP (m^2^)
PBr	86	312	98	79.0	109
PBa	35	253	64	23.3	19
PBa/DQ-1%	32	225	57	20.9	15
PBa/DQ-3%	29	201	51	18.8	10
PBa/DQ-5%	27	172	46	17.1	7

## Data Availability

Not applicable.

## Supplementary Materials

The following supporting information can be downloaded at: https://www.mdpi.com/article/10.3390/ijms24054314/s1.
