# Supplementary figures and images for: Phosphorus-Containing Polybenzoxazine Aerogels with Efficient Flame Retardation and Thermal Insulation
Source: Int J Mol Sci. 2023 Feb 21;24(5):4314. doi: 10.3390/ijms24054314 (PMC10001678; doi:10.3390/ijms24054314)

## Supporting information

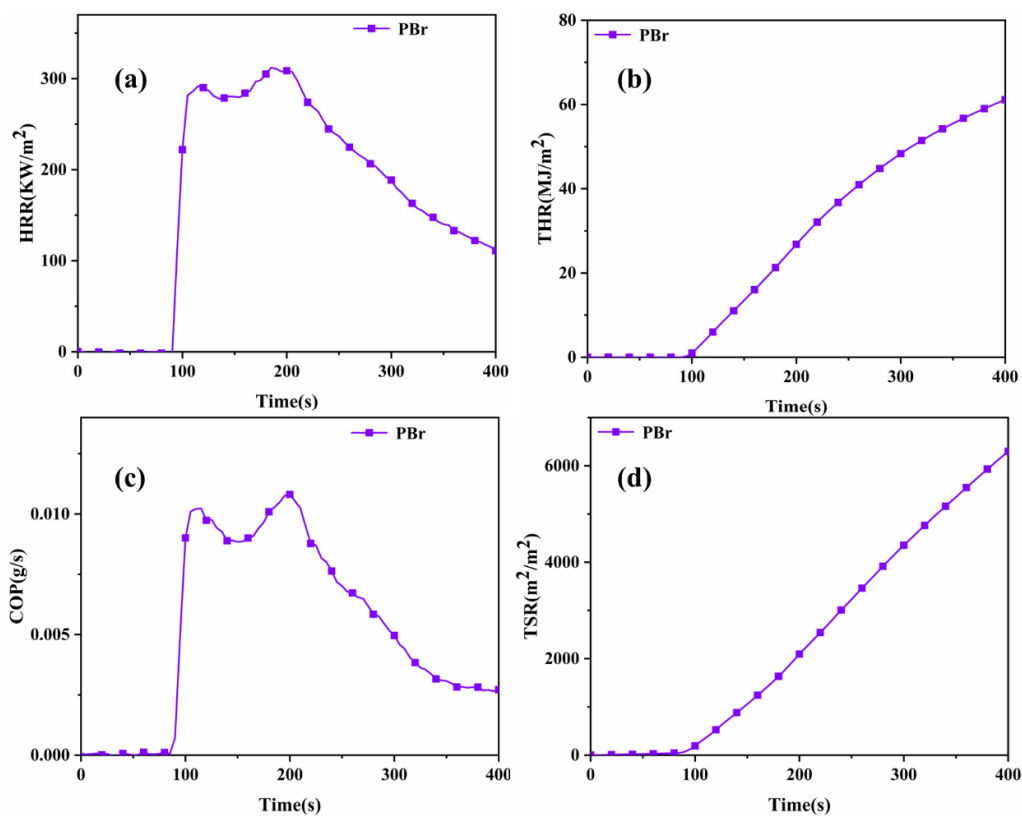

Figure S1. (a) HRR, (b) THR, (c) COP, and (d) TSR versus time curves of PBr.

Supplement: Supplementary file 1 [file ijms-24-04314-s001.zip › ijms-2214246-supplementary.pdf]
